# *Toxoplasma gondii* and multiple sclerosis: a population-based case–control study

**DOI:** 10.1038/s41598-020-75830-y

**Published:** 2020-11-02

**Authors:** Alessandra Nicoletti, Calogero Edoardo Cicero, Loretta Giuliano, Valeria Todaro, Salvatore Lo Fermo, Clara Chisari, Emanuele D’Amico, Vincenza Paradisi, Antonia Mantella, Alessandro Bartoloni, Vito Sofia, Francesco Patti, Mario Zappia

**Affiliations:** 1 Department of Medical and Surgical Sciences and Advanced Technologies “G.F. Ingrassia”, Section of Neurosciences, University of Catania, Via Santa Sofia 78, 95123 Catania, Italy; 2 Italian Society of General Medicine (SIMG), Catania, Italy; 3 Department of Experimental and Clinical Medicine, Infectious and Tropical Diseases Unit, University of Florence, Florence, Italy

**Keywords:** Multiple sclerosis, Parasitic infection

## Abstract

According to the hygiene hypothesis, parasites could have a protective role in the development of Multiple Sclerosis (MS). Our aim was to assess the association between presence of anti-*Toxoplasma gondii* antibodies and MS. MS patients were randomly selected from a population-based incident cohort of MS patients in the city of Catania. Age and sex-matched controls were randomly selected from the general population. Clinical and sociodemographic variables were recorded with a structured questionnaire and a blood sample was taken for serological analysis. Specific *T. gondii* IgG have been detected with a commercial kit. Adjusted Odds Ratios (ORs) were estimated using unconditional logistic regression. 129 MS subjects (66.7% women with a mean age 44.7 ± 11.0 years) and 287 controls (67.3% women with a mean age 48.1 ± 15.6 years) have been enrolled in the study. Anti-*T. gondii* antibodies were found in 38 cases (29.5%) and 130 controls (45.4%) giving an adjusted OR of 0.56 (95%CI 0.34–0.93). History of mononucleosis and high educational level were significantly associated with MS (adjOR 2.22 and 1.70 respectively) while an inverse association was found between high educational level and *T. gondii* seropositivity (adjOR 0.42). Our results further support the protective role of parasitic infections in MS.

## Introduction

Multiple Sclerosis (MS) is an inflammatory disease of the Central Nervous System (CNS) caused by an autoimmune process, characterized by a classical Th1 response^1^*.* MS is considered a multifactorial disease, due to a complex interaction between genetic and environmental factors. The impact of some environmental and lifestyle risk factors has been largely demonstrated, such as smoking, vitamin D levels and Epstein Barr virus (EBV) infection^2^. The role of infective agents is further supported by the “hygiene hypothesis”, that postulates that infectious agents in an earlier age could act as a protective factor^3^. Hygiene hypothesis has been proposed as a possible explanation for the increased incidence of allergy and autoimmune diseases, including MS, in western countries and an imbalance between Th1 and Th2 cells was proposed as an immunological explanation^4^. As a matter of fact, epidemiological studies showed that prevalence of MS has been increasing over the time, especially in countries that have improved their socioeconomic and sanitation levels^5^ probably through a progressive decrease in the prevalence of infections^6^. *Toxoplasma gondii (T. gondii)* is an obligate intracellular protozoan replicating in the mammalian cells with a complex life cycle consisting of a sexual cycle in its feline definitive host and an asexual cycle in its intermediate host (livestock, birds, humans)^7^. It is estimated that at least one third of the general population is infected with *T. gondii* worldwide^7^**.** The risk of being infected with *T. gondii* is associated with a low socio-economic status^8^, the close contact with cats, and with eating raw or undercooked pork and lamb meat of infected hosts^7^. The clinical presentation of *T. gondii* infection is generally asymptomatic in immunocompetent humans, however in immunocompromised hosts it may give rise to more severe clinical manifestations, such as encephalitis^7^.

Several studies have suggested a possible association between chronic *T. gondii* infection and autoimmune diseases such as rheumatoid arthritis and systemic lupus erythematosus^9^, but the association between toxoplasmosis and MS is still unclear^10^.

In fact, a recently published metanalysis of studies assessing the association between MS and *T. gondii* has demonstrated a lower, but still not significant, protective effect of the parasite on the risk of being diagnosed with MS^10^. However, all of the studies included in the meta-analysis have relatively small sample sizes^10^, negatively impacting the statistical power of the studies. In order to overcome these limitations, we carried out a population-based case–control study to better evaluate the relationship between *T. gondii* and MS.

## Materials and methods

### Study population

This study is part of a population-based case–control study conducted by our group in the city of Catania to assess the association between environmental risk factors and MS. The study gathered clinical data and serum samples from 164 MS patients and 481 age and sex matched controls. Background and methods have been extensively reported elsewhere^11^. Briefly, MS patients were randomly selected from an incident cohort of MS patients consisting of all incident cases in the city of Catania who had had the onset of disease from 1st January 1975 to 31st December 2004 and comprises 367 MS patients^12–14^. From this cohort 164 MS patients, diagnosed according to Poser’s criteria^15^ were randomly selected.

Population controls were enrolled in the study through an ‘‘equal probability selection method’’ (EPSEM). At least three control subjects per each MS patient, frequency matched by age and sex were enrolled from the general population of Catania using multistage sampling methods.

Control subjects, not affected by neurological disorders, were randomly selected from General Practitioners’ cabinet list.

### Blood sample collection and analysis

At the time of the enrolment 20 cc of whole blood were taken for each subject. Serum samples were analysed at the laboratories of the Infectious Diseases Institute of the University of Florence and have been analysed by a biologist blinded to the status of the participants. Specific *Toxoplasma gondii* IgG have been detected with a commercial ELISA kit (DIA.PRO Toxoplasma IgG; Diagnostic Bioprobes s.r.l.) with a sensitivity and specificity reported by the manufacturer of 98%.

### Assessment of exposures

A face-to-face semi-structured standardized questionnaire investigating environmental exposures and comorbidities has been administered to all patients and controls. All subjects have been asked the questions directly and in the case of a disability-related impairment in communication, to a proxy responder, preferably the spouse or sibling. Details of the screening instrument are reported elsewhere^11^.

### Statistical analysis

A double entry process was implemented to enter all the gathered clinical, laboratory and demographic variables into the dataset. Data cleaning included range, consistency checks and cross referencing with original data to identify and correct missing values. To describe the variables, frequency, means and standard deviations were used. Variables normally distributed after the Shapiro–Wilk test were analysed with the two tailed t test. Otherwise a Mann–Whitney test was conducted. For qualitative variables significance testing we employed the chi squared and Fisher test. For the prevalence of anti-*T. gondii* antibodies the 95% CI have been calculated. Statistical significance was set at p < 0.05. In order to test the association between positivity to *T. gondii* antibodies and explanatory variables we conducted an unconditional logistic regression analysis (two-tailed, α = 0.05). Variables associated with a p ≤ 0.25 were then used to compute the multivariate model. We manually compared the log-likelihood of the model with and without a specific variable with the likelihood ratio test. Sex and age have been considered a priori confounder variables.

The study has been performed in accordance with STROBE (Strengthening The Reporting of Observational Studies in Epidemiology) guidelines (Supplementary methods 1). All the analyses have been conducted with the software STATA 12.0.

### Ethics

All the patients and controls enrolled in the study have been given a paper briefly explaining the reason of the study and have been resumed orally by the GP or the neurologist. A written informed consent model has then been signed by the patient or the control or a legal representative in the case of severe disability if they accepted to participate in the study. The study has been approved by the Ethical Committee of the AOU Policlinico Vittorio Emanuele with the code 64/2018/PO.

## Results

The original sample of the case–control study consisted of 164 cases and 481 controls. Baseline characteristics (sex, age at onset, disease form and expanded disability status scale) of the 164 enrolled patients were not significantly different from the whole MS population (incident cohort of MS patients consisting of 367 patients)^11^. Out of the 164 MS patents a serum sample was available for 129 (78.6%) cases and 287 (59.6%) controls.

At the time of the enrolment, MS patients had a mean age of 44.7 ± 11.0 years, and 86 (66.7%) were women; mean age of control subjects was 48.1 ± 15.6 while 193 were women (67.3%). Mean age at onset was 32.7 ± 11.3 years with a mean disease duration of 12.0 ± 8.2 years. The most frequent type of MS was the RR form (n = 103; 79.8%) and the mean expanded disability status scale (EDSS) at the enrolment was 2.5 ± 2.2. The majority of patients was under treatment with an immunomodulating drug (n = 76; 59.8%) while only 17 (13.4%) were not taking any therapy, as reported in Table 1.

**Table 1:** Demographic characteristics of Multiple Sclerosis patients.

	Multiple Sclerosis patients (n = 129)
**Age, y (mean ± SD)**	44.7 ± 11.0
**Sex (women), n (%)**	86 (66.7)
**Age at onset, y (mean ± SD)**	32.7 ± 11.3
**Disease duration, y (mean ± SD)**	12.0 ± 8.2
**Disease type, n (%)**	
Relapsing Remitting	103 (79.8)
Secondary Progressive	23 (17.0)
Primary Progressive	2 (1.5)
Other	2 (1.5)
**EDSS, mean ± SD**	2.5 ± 2.2
**Treatment, n (%)**	
No therapy	17 (13.4)
Immunomodulators	76 (59.8)
Immunosuppressants	20 (15.7)
Monoclonal antibodies	14 (11.0)
***T. gondii *seropositivity, n (%)**	38 (29.5)

n, number; SD, standard deviation; EDSS, expanded disability status scale.

Presence of anti-*T. gondii* antibodies was detected in 38 patients giving a seroprevalence of 29.5% (95%CI 21.8–38.1) and 130 controls resulting in a seroprevalence of 45.4% (95%CI 39.6–51.4) giving an OR of 0.50 (95%CI 0.32–0.78; *p-value* = 0.002).

We have also previously assessed the presence of antibodies anti *Toxocara canis (T. canis)*^16^. Eleven MS cases (8.5%) and 23 (8.0%) controls presented antibodies anti *T. canis*. Four MS cases (3.1%) and 14 (4.9%) controls presented a dual infection and were seropositive for both *T. canis* and *T. gondii.* Presence of dual infection was not significantly associated with MS at univariate analysis (OR 0.62; 0.20–1.93; *p-value* 0.4).

At univariate analysis MS was strongly associated with education with a significant trend (chi square 23.5; *p-value* 0.0003) reaching a strongest positive association for university degree (OR 6.87; 95%CI 2.90–16.20; *p-value* < 0.0001); MS was also positively associated with history of mononucleosis and inversely associated with history of autoimmune diseases as reported in Table 2.

**Table 2:** Univariate and multivariate analysis.

	Multiple Sclerosis patients (n = 129)	Controls (n = 287)	Univariate analysis			Multivariate analysis		
			OR	95% CI	p-value	adjOR	95% CI	p-value
**Age, y (mean ± SD)**	44.7 ± 11.0	48.1 ± 15.6	0.98	0.97- 1.00	**0.03**	0.99	0.98–1.01	0.6
**Sex (women), n (%)**	86 (66.7)	193 (67.3)	0.97	0.62–1.51	0.9	1.07	0.66–1.72	0.8
**Profession, n (%)**								
Professional/Office job	51 (46.5)	90 (31.8)	1	*/*	*/*			
Industry	6 (5.4)	39 (13.8)	0.27	0.11–0.68	**0.006**			
Tradesman	4 (3.6)	9 (3.2)	0.78	0.23–2.67	0.7			
Artisan	3 (2.7)	7 (2.5)	0.75	0.18–3.05	0.7			
Agricolture	/	1 (0.3)	/	/	/			
Housewife	25 (22.7)	79 (27.9)	0.56	0.32–0.98	**0.04**			
Unemployed	9 (8.1)	10 (3.5)	1.58	0.61- 4.16	0.5			
Other	12 (10.9)	48 (17.0)	0.44	0.21–0.97	**0.03**			
**Number of siblings (mean ± SD)**	2.97 ± 2.0	3.5 ± 2.9	0.92	0.83–1.001	0.08			
**Education, n (%)**								
Illiterate/primary school	8 (6.2)	66 (23.0)	1	/	**/**			
Secondary school	38 (29.5)	94 (32.7)	3.33	1.46–7.60	**0.004**			
High school	48 (37.2)	85 (29.6)	4.66	2.06–10.5	** < 0.0001**			
University	35(27.1)	42 (14.6)	6.87	2.90–16.25	** < 0.0001**			
**Years of schooling, n (%)**								
≤ 8 years	46 (35.7)	160(55.7)	1	/	**/**	1	/	**/**
* ≥ *9 years	83 (64.3)	127 (44.2)	2.27	1.48–3.49	** < 0.0001**	1.70	1.07–2.73	**0.02**
**Cat ownership, n (%)**	41 (31.8)	92 (32.1)	0.99	0.63–1.54	0.9			
**Dog ownership, n (%)**	63 (48.8)	135 (47.0)	1.07	0.71–1.63	0.7			
***T. gondii*** **positivity, n (%)**	38 (29.5)	130(45.4)	0.50	0.32–0.78	**0.002**	0.56	0.34–0.93	**0.02**
***T. canis*** **positivity, n (%)**	11 (8.5)	23 (8.0)	1.07	0.50–2.27	0.2			
***T. gondii* and *T. canis *positivity, n (%)**	4 (3.1)	14 (4.9)	0.62	0.20–1.93	0.4			
**Mononucleosis, n (%)**	22 (17.0)	20 (7.1)	2.70	1.42- 5.16	**0.003**	2.22	1.12–4.39	**0.02**
**Autoimmune diseases, n (%)**	6/129 (4.6)	33/269 (12.3)	0.34	0.14–0.85	**0.02**	0.32	0.12–0.81	**0.02**
**Smoking** **(Never/Ever), n (%)**	56/107 (52.3)	127 (44.4)	1.11	0.80–1.53	0.5			

Significant p values are highlighted in bold.

n, number; SD, standard deviation; OR, odds ratio; CI, confidence intervals; adjOR, Adjusted OR.

At the multivariate analysis, after adjusting for age and sex considered as a priori confounders, a significant negative association was found between MS and *T. gondii* seropositivity (adjOR 0.56; 0.34–0.93; *p-value* 0.02) and history of autoimmune diseases (adjOR 0.32; 0.12–0.81; *p-value* 0.02), while a positive association was found with years of schooling (adjOR 1.70; 1.07–2.73; *p-value* 0.02) and history of mononucleosis (adjOR 2.22; 1.12–4.39; *p-value* 0.02). Forcing occupation in the final model did not modify the strength of the association with *T. gondii* (adjOR 0.56; 0.32–0.97; *p-value* 0.04).

Both at univariate and multivariate analysis (adjusting by age and sex respectively) *T. gondii* sero-positivity was significantly associated with age (adjOR 1.06; 95%CI 1.05–1.08; *p-value* < 0.0001) and female sex (1.97; 95%CI 1.04–1.08; *p-value* < 0.0001), but not with the presence of cats or other domestic animals (dogs, birds). Furthermore, a strong negative association was found between education and *T. gondii* seropositivity (years of schooling ≥ 9 years) with an adjOR of 0.42 (95%CI 0.27–0.65; *p-value* < 0.0001).

Considering only the control population 33 subjects (12.3%) out of 269 reported a positive history for autoimmune diseases other than MS (25 autoimmune thyroiditis, seven rheumatoid arthritis and one LES). We found a negative association, even if borderline significant, between *T. gondii* seropositivity and presence of autoimmune diseases (0.54; 95%CI 0.25–1.16; *p-value* 0.1). This association was stronger and significant when adjusting by age and sex (adj OR 0.38; 95%CI 0.16–0.90; *p-value* 0.03) (Supplementary Table S1).

## Discussion

We found a negative association between *T. gondii* and MS, suggesting a possible protective role of this parasite and supporting the hygiene hypothesis. According to the hygiene hypothesis, in fact, low exposure to pathogens early in life can increase the risk for autoimmune diseases, including MS and allergy, especially in western countries probably due to an immunological imbalance between Th1 and Th2 response^4^***.***

However, parasites are divided into two major groups, helminths and protozoans, that can act with different immunological mechanisms. Helminths, in fact, induce a Th2 response characterized by the production of IL-4, IL-5, and IL-10^17^ while protozoans induce a very strong Th1 response by the production of pro-inflammatory mediators, such as IL-12, IFN- γ, and nitric oxide^18^.

Helminths modulation of autoimmune response has been shown in different autoimmune diseases^19–21^. In MS, the protective influence of helminths is mediated by the down modulation of inflammatory responses and enhancement of immune regulation^22^. Some experimental and epidemiological evidences have supported this hypothesis. In particular, infection with *Schistosoma mansoni* has demonstrated to exert a protective effect on the development of Experimental Allergic Encephalomyelitis (EAE) in infected mice reducing IFN-γ, tumor necrosis factor alpha (TNF-α) and IL-12 production and dampening the classical Th1 response^23^. These effects are thought to be associated with the immunological switch to a Th2 response caused by parasite infestation^24^.

Apart from helminths, recently interest has been drawn to *T. gondii*, an intracellular parasite that causes toxoplasmosis in both humans and animals. Several studies have suggested an association between chronic *T. gondii* infection and autoimmune diseases such as diabetes mellitus type I, rheumatoid arthritis and systemic lupus erythematosus**,** but conflicting results have been reported, suggesting that *T. gondii* may play a positive or negative role on different autoimmune conditions^9,25,26^*.*

In the last years some studies have also investigated the association between toxoplasmosis and MS. To the best of our knowledge up to date only five hospital-based case–control studies^26–30^ have been carried out and all, except one^28^, reported a negative association*,* even if it was significant only in two studies^27,29^. Nonetheless it should be noted that for the majority of these studies sample size was very small, thus the lack of significance may be also attributable to a lack of adequate power. Only one study^28^ reported a positive, but not significant, association between *T. gondii* infection and MS, but also in this case sample size was particularly small (52 MS patients and 45 controls)^28^**.** All these previously conducted studies have been included in a recent metanalysis (669 MS patients and 770 controls)^10^ where a lower pooled seroprevalence of *T. gondii* was recorded among MS patients (32.4% versus 39.1%) leading to a negative, even if not significant, association with a combined OR of 0.72 (95% CI: 0.49–1.06)^10^***.***

Our study represents one of the largest case–control studies aimed to assess the possible association between *T. gondii* and MS and the only one with a population-based design, avoiding possible selection bias. On average, seroprevalence detected among cases (29.5%) and controls (45.4%) was close to those reported in literature^10^ with a significant negative association between *T. gondii* seropositivity and MS with an OR of 0.50.

It should be underlined that, in agreement with literature data, history of mononucleosis was positively associated with MS^22^. On the other hand, along with *T. gondii* seropositivity, in our sample also positive history for other autoimmune diseases was negatively associated with MS suggesting a possible protective effect. Even if some studies suggest an increased frequency of some autoimmune disease among MS patients the possible relationship between MS and other autoimmune diseases is still debated^31^. However, it should be noted that the presence of autoimmune disease in our study was based just on clinical history, thus this finding should be interpreted with caution.

Considering the *T. gondii* seropositivity, in agreement with literature, age and lower educational level represented a possible risk factor^8,32^, while we did not find any significant association between pet ownership and *T. gondii* seropositivity. Furthermore, MS was associated with higher levels of education with a significant trend, reaching an OR of 6.87 for those who attended the university^33,34^. Conversely higher levels of education were associated with lower prevalence of *T. gondii* antibodies. Education is a proxy of the socioeconomic status that is generally associated with increased levels of sanitation, thus with a reduced exposition to different pathogens during childhood. This evidence is also in agreement with the hygiene hypothesis, as highlighted by some initial MS studies performed in the 90s^33,34^.

Our findings provide a further support for the hygiene hypothesis. However, as for other protozoans, it should be noted that *T. gondii* induces a very strong Th1 response rather than a characteristic Th2 response observed for helminths^35^. As a matter of fact literature data suggest that a possible protective role of *T. gondii* could be exerted through an increased production of IL-10^35^. Indeed, studies using both human and animal models have suggested a number of different underlying mechanisms. In particular in animal models it has been demonstrated that *T. gondii* not only induces the production of IFN-γ in T-bet + T helper cells, but also of IL-10, indicating that the parasite elicits regulatory activities in its acute and chronic phase^9,35^***.*** According to literature IL-10 could play a relevant role in reducing autoimmune reaction in MS^36^***, ***thus, as for helminth models, it is possible to hypothesize that *T. gondii* could exert a protective effect through the increase of IL-10 levels.

Moreover, host response to toxoplasmosis acute infection is characterized by a strong inflammatory activation mediated by the Th17 CD4 lymphocytes producing IL-17^37^. In turn, *T. gondii* has developed survival mechanisms that allow for the parasite survival through the dampening of the Th-17 response via the induction of IL-27 production by antigen presenting cells^37^. Interestingly, high levels of IL-27 have been demonstrated in cerebrospinal fluid of MS patients probably exerting an antinflammatory activity^38^.

Another possible explanation of the immunosuppressive activity of *T. gondii* infection can be related to the activation of the FoxP3+ T cells, which dampen the immune system response during chronic *T. gondii* infection probably by reducing the availability of IL-2, a strong proinflammatory cytokine^39^. Lastly, also TGF-beta is relevant in the immunosuppression present during the chronic *T. gondii* infection^40^, a cytokine that plays a major role in the promotion of CD4 regulatory activity in MS patients^41^.

Nonetheless, to date, no data are available on experimental infection of *T. gondii* in EAE mice, the mouse model of MS, thus studies are needed to understand the underlying pathophysiological mechanisms**.**

In the attempt to understand the possible underlining mechanisms that could explain the relationship between parasitic infections and autoimmune diseases, in a previous study involving the same sample of cases and controls we evaluated the association between MS and *T. canis*, one of the most prevalent helminthiases worldwide, but we did not find any significant association^16^.

From a methodological point of view our studies presents many strengths. This is the first population-based case–control study carried out to evaluate the possible association between MS and *T. gondii.* MS patients, in fact, were randomly enrolled from a well-established cohort of incident MS patients, while controls were selected from the general population using a multistage sampling method^11^, thus reducing the risk of a possible selection bias among cases. Furthermore, along with the study carried out by Stascheit et coll. in 2015^27^, our study represents the largest case–control study carried out to investigate the possible relationship between MS and *T. gondii*.

Along with selection bias, recall bias represents the second most common type of bias affecting case–control studies. However, in our study the possible role of recall bias can be ruled out since exposition to *T. gondii* has been assessed by the detection of serum IgG antibodies and the biologist who performed the serological assessment was blinded with respect to the disease status.

Nonetheless the retrospective nature of the case–control design largely limits the interpretation of our results. We cannot exclude the role of a low socioeconomic status in modulating the interaction between *T. gondii* infection and MS considering that it acts as a risk factor for *T. gondii* and as protective factor for MS. However, we have conducted a multivariate model including the level of education and occupational status considered both proxies of socioeconomic status and we found a still significant protective effect of *T. gondii* on MS. Moreover, the role of eating raw/undercooked meat and its relation to higher risk of *T. gondii,* was not explored due to the lack of this information on the original case–control study. At any rate, while there is an increased risk of infection with *T. gondii* for consuming raw/undercooked meat^7^, the association between meat consumption and MS is still unclear^42^.

Indeed we are unable to establish the exact sequence of the events, thus we cannot exclude that the exposition to *T. gondii* has been occurred after the disease onset. Nonetheless it should be underlined that a previous study found no association between disease duration and antibodies load^29^.

In conclusion even if our study supports the possible protective role of *T. gondii* on MS, further clinical and experimental studies are needed to confirm our results and to understand the underlying pathophysiological mechanisms^27^*.*

## Supplementary information

Supplementary Information 1.

Supplementary Information 2.

## Data Availability

The datasets generated during and/or analysed during the current study are available from the corresponding author on reasonable request.

## References

[1] Hemmer B, Kerschensteiner M, Korn T (2015). Role of the innate and adaptive immune responses in the course of multiple sclerosis. Lancet Neurol..

[2] Olsson T, Barcellos LF, Alfredsson L (2017). Interactions between genetic, lifestyle and environmental risk factors for multiple sclerosis. Nat. Rev. Neurol..

[3] Correale J, Farez MF, Gaitán MI (2017). Environmental factors influencing multiple sclerosis in Latin America. Mult. Scler. J. Exp. Transl. Clin..

[4] Stiemsma LT, Reynolds LA, Turvey SE, Finlay BB (2015). The hygiene hypothesis: current perspectives and future therapies. Immunotargets Ther..

[5] Cabre P (2005). Role of return migration in the emergence of multiple sclerosis in the French West Indies. Brain.

[6] Fleming JO, Cook TD (2006). Multiple sclerosis and the hygiene hypothesis. Neurology.

[7] Tenter AM, Heckeroth AR, Weiss LM (2000). Toxoplasma gondii: from animals to humans. Int. J. Parasitol..

[8] Mareze M (2019). Socioeconomic vulnerability associated to Toxoplasma gondii exposure in southern Brazil. PLoS ONE.

[9] Fischer S (2013). Toxoplasma gondii: bystander or cofactor in rheumatoid arthritis. Immunol. Res..

[10] Saberi R (2018). Is Toxoplasma gondii playing a positive role in multiple sclerosis risk? A systematic review and meta-analysis. J. Neuroimmunol..

[11] Nicoletti A (2016). Risk factors in multiple sclerosis: a population-based case–control study in Sicily. Background and methods. Neurol. Sci..

[12] Nicoletti A (2001). Prevalence and incidence of multiple sclerosis in Catania, Sicily. Neurology.

[13] Nicoletti A (2005). Possible increasing risk of multiple sclerosis in Catania, Sicily. Neurology.

[14] Nicoletti A (2011). Increasing frequency of multiple sclerosis in Catania, Sicily: a 30-year survey. Mult. Scler..

[15] Poser CM (1983). New diagnostic criteria for multiple sclerosis: guidelines for research protocols. Ann. Neurol..

[16] Cicero CE (2020). Lack of association between *Toxocara canis* and multiple sclerosis: a population-based case-control study. Mult. Scler..

[17] Jackson JA, Friberg IM, Little S, Bradley JE (2009). Review series on helminths, immune modulation and the hygiene hypothesis: immunity against helminths and immunological phenomena in modern human populations: coevolutionary legacies?. Immunology.

[18] Munoz M, Liesenfeld O, Heimesaat MM (2011). Immunology of *Toxoplasma gondii*. Immunol. Rev..

[19] Zaccone P, Hall SW (2012). Helminth infection and type 1 diabetes. Rev. Diabet. Stud..

[20] Elliott DE, Weinstock JV (2012). Helminth-host immunological interactions: prevention and control of immune-mediated diseases. Ann. N. Y. Acad. Sci..

[21] Versini M (2015). Unraveling the Hygiene Hypothesis of helminthes and autoimmunity: origins, pathophysiology, and clinical applications. BMC Med..

[22] Correale J, Gaitán MI (2015). Multiple sclerosis and environmental factors: the role of vitamin D, parasites, and Epstein-Barr virus infection. Acta Neurol. Scand..

[23] La Flamme AC, Ruddenklau K, Bäckström BT (2003). Schistosomiasis decreases central nervous system inflammation and alters the progression of experimental autoimmune encephalomyelitis. Infect. Immun..

[24] Sewell D (2003). Immunomodulation of experimental autoimmune encephalomyelitis by helminth ova immunization. Int. Immunol..

[25] Krause I (2009). Anti-infectious antibodies and autoimmune-associated autoantibodies in patients with type I diabetes mellitus and their close family members. Ann. N. Y. Acad. Sci..

[26] Shapira Y (2012). Prevalence of anti-Toxoplasma antibodies in patients with autoimmune diseases. J. Autoimmun..

[27] Stascheit F, Paul F, Harms L, Rosche B (2015). Toxoplasma gondii seropositivity is negatively associated with multiple sclerosis. J. Neuroimmunol..

[28] Oruç S (2016). Relationship of *Toxoplasma gondii* exposure with multiple sclerosis. Eur J. Gen. Med..

[29] Koskderelioglu A, Afsar I, Pektas B, Gedizlioglu M (2017). Is Toxoplasma gondii infection protective against multiple sclerosis risk?. Mult. Scler. Relat. Disord..

[30] Pestehchian N (2014). Frequency of blood-tissue parasitic infections in patients with multiple sclerosis, as compared to their family members. Int. J. Prev. Med..

[31] Marrie RA (2015). A systematic review of the incidence and prevalence of autoimmune disease in multiple sclerosis. Mult. Scler..

[32] Wang T (2018). Seroprevalence of *Toxoplasma gondii* infection in blood donors in mainland China: a systematic review and meta-analysis. Parasite.

[33] Kurtzke JF, Page WF (1997). Epidemiology of multiple sclerosis in US veterans: VII. Risk factors for MS. Neurology.

[34] Hammond SR, McLeod JG, Macaskill P, English DR (1996). Multiple sclerosis in Australia: socioeconomic factors. J. Neurol. Neurosurg. Psychiatry.

[35] Jankovic D (2007). Conventional T-bet(+)Foxp3(−) Th1 cells are the major source of host-protective regulatory IL-10 during intracellular protozoan infection. J. Exp. Med..

[36] Correale J, Farez M, Razzitte G (2008). Helminth infections associated with multiple sclerosis induce regulatory B cells. Ann. Neurol..

[37] Stumhofer JS (2006). Interleukin 27 negatively regulates the development of interleukin 17-producing T helper cells during chronic inflammation of the central nervous system. Nat. Immunol..

[38] Lalive PH (2017). Increased interleukin-27 cytokine expression in the central nervous system of multiple sclerosis patients. J. Neuroinflam..

[39] Tenorio EP, Fernández J, Castellanos C, Olguín JE, Saavedra R (2011). CD4+ Foxp3+ regulatory T cells mediate *Toxoplasma gondii*-induced T-cell suppression through an IL-2-related mechanism but independently of IL-10. Eur. J. Immunol..

[40] Zare-Bidaki M (2016). TGF-β in toxoplasmosis: friend or foe?. Cytokine.

[41] Lee PW, Severin ME, Lovett-Racke AE (2017). TGF-β regulation of T cells in multiple sclerosis. Eur. J. Immunol..

[42] Bagur MJ (2017). Influence of diet in multiple sclerosis: a systematic review. Adv. Nutr..

