# Supplementary material for: Toxoplasma gondii and multiple sclerosis: a population-based case–control study
Source: Sci Rep. 2020 Nov 2;10:18855. doi: 10.1038/s41598-020-75830-y (PMC7606604; doi:10.1038/s41598-020-75830-y)
Supplement: Supplementary file 1 — Supplementary Information 1. [file 41598_2020_75830_MOESM1_ESM.docx]

***Toxoplasma gondii* and Multiple Sclerosis: a population-based case-control study.**

**Alessandra Nicoletti ^1a^*, Calogero Edoardo Cicero^1a^, Loretta Giuliano^1^, Valeria Todaro^1^, Salvatore Lo Fermo^1^, Clara Chisari^1^, Emanuele D’Amico^1^, Vincenza Paradisi^2^, Antonia Mantella^3^, Alessandro Bartoloni^3^, Vito Sofia^1^, Francesco Patti^1^ & Mario Zappia^1^.**

^1^Department of Medical and Surgical Sciences and Advanced Technologies “G.F. Ingrassia”, Section of Neurosciences, University of Catania, Catania, Italy

^2^Italian Society of General Medicine (SIMG), Catania, Italy

^3^Department of Experimental and Clinical Medicine, Infectious and Tropical Diseases Unit, University of Florence, Florence, Italy

**^a^ These authors contributed equally**

**Supplementary Table S1.** Association between autoimmunity and *T.gondii* seropositivity.

|  | **Presence of an autoimmune disease (n=33)** | **No autoimmune diseases (n=236)** | **OR** | **95%CI** | **p-value** | **adjOR** | **95%CI** | **p-value** |
| --- | --- | --- | --- | --- | --- | --- | --- | --- |
| **Age** | 47.8±14.9 | 48.2±15.4 | 0.99 | 0.97-1.02 | 0.862 | 1.00 | 0.98-1.03 | 0.502 |
| **Sex (women)** | 32 (97%) | 152 (64.4) | 17.7 | 2.4-131.7 | 0.005 | 20.7 | 2.75-155.38 | **0.003** |
| ***T. gondii* seropositivity** | 11 (33.3%) | 113 (48.1%) | 0.53 | 0.25-1.16 | 0.115 | 0.38 | 0.16-0.90 | **0.03** |

**Legend**: OR, Odds Ratio; adjOR, adjusted Odds Ratio.
